# Control of Zn uptake in *Arabidopsis halleri*: a balance between Zn and Fe

**DOI:** 10.3389/fpls.2013.00281

**Published:** 2013-07-31

**Authors:** Varanavasiappan Shanmugam, Jing-Chi Lo, Kuo-Chen Yeh

**Affiliations:** Agricultural Biotechnology Research Center, Academia Sinica TaipeiTaiwan, Republic of China

**Keywords:** *Arabidopsis halleri*, hyperaccumulators, ZIP transporters, Fe homeostasis, Zn tolerance

## Abstract

Zinc (Zn) is an essential plant micronutrient but is toxic in excess. To cope with excess Zn, plant species possess a strict metal homeostasis mechanism. The Zn hyperaccumulator *Arabidopsis halleri* has developed various adaptive mechanisms involving uptake, chelation, translocation and sequestration of Zn. In this mini review, we broadly discuss the different Zn tolerance mechanisms and then focus on controlled Zn uptake in *A. halleri*. Members of the ZRT/IRT-like protein (ZIP) family of metal transporters are mainly regulated by Zn and are involved in Zn uptake. A few members of the ZIP family, such as IRT1 and IRT2, are regulated by iron (Fe) and can transport multi-metals, including Zn, Fe, Mn, Cd, and Co. This mini-review also discusses the differential expression of multiple metal ZIP transporters in *A. halleri* and *A. thaliana*, a non-hyperaccumulator, with Zn exposure as well as Fe deficiency and their role in controlled Zn uptake and tolerance.

## INTRODUCTION

Heavy metal pollution in the soil has greatly increased over the past decades because of mining and industrial activities, overuse of chemical fertilizers, and waste-water irrigation (Nriagu and Pacyna, 1988). Metals such as cadmium (Cd), mercury (Hg), and lead (Pb) are considered non-essential because they do not have any role in any physiological process in plants. In contrast, metals such as zinc (Zn), iron (Fe), copper (Cu), manganese (Mn), molybdenum (Mo), and nickel (Ni) are essential micronutrients required for normal growth and metabolism of plants (Marschner, 1995). For example, Zn is a cofactor for many enzymes, and many proteins contain Zn-binding structural domains (Clarke and Berg, 1998; Guerinot and Eide, 1999).

Zn has an important role in several physiological and metabolic processes in plants (Ramesh et al., 2004). However, in excess, Zn can be toxic and influence the status of the other metal ions, thus resulting in severe growth defects in plants (Marschner, 1995). At toxic concentrations, Zn replaces other divalent cations such as Fe, magnesium (Mg) and Mn, which are involved in the proper functioning of a number of photosynthetic enzymes, thereby resulting in lower photosynthetic rates and photo-oxidative damage (Vanassche and Clijsters, 1986a,b, 1990). To avoid potential toxicity caused by displacement of these elements, metal ion homeostasis must be strictly controlled in plants.

Zn-tolerant and -hyperaccumulating species have various mechanisms to cope with excess Zn levels. This review focuses on the current understanding of the Zn homeostasis network in Zn hyperaccumulators and addresses Zn and Fe crosstalk in response to Zn tolerance and hyperaccumulation.

## Zn HYPERACCUMULATORS: A MODEL SYSTEM TO UNDERSTAND Zn HOMEOSTASIS IN PLANTS

Plant species that can grow at growth-limiting concentrations of metals such as Ni, Zn, Cd, Co, or Cu have naturally selected increased tolerance and are called metal-tolerant species. In addition, a few plant species, called as metal hyperaccumulators, can tolerate and also accumulate these metals in their shoot tissues at four orders of magnitude higher than those of non-hyperaccumulators (Roosens et al., 2008; Kramer, 2010). Approximately 500 plant taxa can accumulate such high concentrations of potentially toxic metals; 20 of these are Zn hyperaccumulators (Baker and Brooks, 1989; Reeves and Baker, 2000; Kramer, 2010).

Several species of the Brassicaceae family are metal hyperaccumulators. Examples are *Noccaea caerulescens *and *Arabidopsis halleri. N. caerulescens* was the first identified Zn hyperaccumulator and was reported to accumulate about 25,000 to 30,000 μg g^-^^1 ^dry weight (DW) of total Zn (Brown et al., 1995; Shen et al., 1997). *A. halleri* is a Zn/Cd hyperaccumulator (Ernst, 1974; Kupper et al., 2000; Zhao et al., 2000, 2006; Bert et al., 2002; Cosio et al., 2004) and can accumulate >10,000 and >100 μg g^-^^1^ DW of Zn and Cd, respectively. *A. halleri* is closely related to *A. thaliana*, a Zn non-hyperaccumulator, whose genome has been thoroughly explored. Thus, *A. halleri *is a good model system to study Zn tolerance and Zn hyperaccumulation mechanisms. A few comparative transcriptomic studies of *A. halleri *have identified several key genes involved in the Zn hyperaccumulation process (Becher et al., 2004; Weber et al., 2004; Chiang et al., 2006; Talke et al., 2006).

## STRATEGIES OF Zn TOLERANCE AND HYPERACCUMULATION: SEQUESTRATION FOR DETOXIFICATION

Zn hyperaccumulators prevent toxicity symptoms and cope with excess metal ions using various strategies such as effective metal uptake, increased xylem loading and increased detoxification in shoot tissues (Kramer, 2010). In recent years, many components involved in these processes have been identified and characterized (Verbruggen et al., 2009; Kramer, 2010; Deinlein et al., 2012). Zn tolerance and hyperaccumulation are better understood because of investigation of species closely related to the model *A. thaliana*. *A. halleri *and *N. caerulescens *share 94 and 88% nucleotide similarity, respectively, with *A. thaliana*. This similarity paves the way for detailed transcriptomic studies and proteomic profiling with respect to deficiency or excess Zn (Becher et al., 2004; Weber et al., 2004; Chiang et al., 2006; Talke et al., 2006; van de Mortel et al., 2006; Schneider et al., 2012). These studies have provided knowledge of Zn uptake, xylem loading and unloading in the detoxification process and have shed light on the involvement of other metal homeostasis networks in Zn uptake and tolerance mechanisms.

Zn hyperaccumulators possess effective root-to-shoot Zn translocation mechanisms through symplastic movement and effective xylem loading (Clemens, 2006; Kramer, 2010; Verbruggen et al., 2009). In recent years, several types of transporters involved in this process have been identified in Zn hyperaccumulators and thoroughly investigated. P-type ATPase (HMA) transporters are mainly involved in root-to-shoot translocation of Zn (Hussain et al., 2004; Verret et al., 2004; Hanikenne et al., 2008; Kim et al., 2009; Barabasz et al., 2010; Lochlainn et al., 2011). *HMA4* is triplicated and also constitutively expressed at a high level in *A. halleri*, thereby mediating effective root-to-shoot translocation and resulting in Zn tolerance (Hanikenne et al., 2008). However, overexpressing *AhHMA4* in *A. thaliana* did not considerably enhance root-to-shoot translocation of Zn and caused Zn hypersensitivity because of lack of an efficient detoxification mechanism in shoot tissues (Hanikenne et al., 2008). These observations emphasize the complexity of metal hyperaccumulation and tolerance mechanisms of metal hyperaccumulators. In addition to P-type ATPases, members of multi-drug and toxic compound extrusion transporters (MATEs) and oligopeptide transporters are highly and constitutively expressed in Zn hyperaccumulators and reported to be involved in Zn translocation (Talke et al., 2006; van de Mortel et al., 2006; Hu et al., 2012; Pineau et al., 2012). FRD3, a member of the MATE family, which functions in citrate efflux into the root vasculature and is involved in the long-distance transport of Fe, was more highly expressed in *A. halleri* than in *A. thaliana*. Recently, FRD3 was found to play a role in Zn tolerance in *A. thaliana* and, possibly, Zn translocation (Pineau et al., 2012). Once Zn is efficiently translocated into shoot tissues, several tonoplast transporters participate in the sequestration of Zn into shoot vacuoles. Metal tolerance protein 1, a tonoplast-localized Zn transporter, is highly expressed in both roots and shoots of *A. halleri *and also linked to a major quantitative trait locus (QTL) responsible for Zn tolerance (Drager et al., 2004; Kobae et al., 2004; Gustin et al., 2009; Kawachi et al., 2009; Shahzad et al., 2010; Willems et al., 2010). Some other members of HMA and ATP-binding cassette transporters are highly expressed in shoots of *A. halleri*, but their exact role in vacuole sequestration of Zn has not been proven by functional studies (Becher et al., 2004; Weber et al., 2004; Chiang et al., 2006).

## ADDITIONAL UPTAKE CONTROLS: Fe HOMEOSTASIS AND Zn TOLERANCE

Zn enters the root system through specific membrane transporters, mainly ZRT/IRT-like protein (ZIP) transporters. The Arabidopsis genome contains 15 members of the ZIP family (**Table 1**). Most are located in the plasma membrane and are involved in micronutrient uptake. IRT1 is well characterized and regulated by Fe status. IRT1 can transport Fe, Mn, Co, Cd, and Zn. The knockout mutant of *IRT1*, *irt1-1*, exhibits severe growth defects, and excess supply of Fe can rescue the defective growth (Vert et al., 2002). IRT2, too, is regulated by Fe status and can transport both Fe and Zn (Vert et al., 2001, 2009). Under Fe deficiency, FIT, together with AtbHLH38 and AtbHLH39, members of the basic helix-loop-helix transcription factor family, transcriptionally regulate the expression of *IRT1 *and *IRT2* (Yuan et al., 2008; Wu et al., 2012). Promoter regions of *IRT1* and *IRT2* contain the E-box motif CANNTG, a potential binding site for FIT (Colangelo and Guerinot, 2004). Apart from IRT1 and IRT2, other ZIP family members are mainly regulated by Zn status and are involved in Zn transport (**Table 1**). Under Zn deficiency, 2 members of the basic-region leucine-zipper family of transcription factors, bZIP19 and bZIP23, are involved in the transcriptional regulation of ZIP family transporters by binding ZDRE elements in their promoter region (Assunção,et al., 2010). In light of the ability of the ZIP family transporters to conduct multi-metal transport, their expression and regulation under excess Zn or other metal ions is likely a complex phenomenon.

**Table 1:** Expression and transport properties of ZRT/IRT-like protein (ZIP) transporters.

Transporter	Regulated by	Metal transport	Expression (Ah to At )	Related reports
IRT1	Fe, Zn	Zn, Fe, Mn, Cd, Co	<	Eide et al. (1996), Korshunova et al. (1999), Guerinot (2000), Vert et al. (2002), Connolly et al. (2002), Shanmugam et al. (2011)
IRT2	Fe, Zn	Fe, Zn	<	Guerinot (2000), Vert et al. (2002), Shanmugam et al. (2011)
IRT3	Fe, Zn	Fe, Zn	<	Guerinot (2000), Chiang et al. (2006), Talke et al. (2006), Lin et al. (2009), Assunção (2010)
ZIP1	Zn	Zn, Mn	<	Grotz et al. (1998), Guerinot (2000), Talke et al. (2006), Assunção (2010), Milner et al. (2013)
ZIP2	Zn	Zn, Mn1	–	Grotz et al. (1998), Guerinot (2000), Wintz et al. (2003), Yang et al. (2010), Milner et al. (2013)
ZIP3	Zn	Zn, Fe, Mn	<	Grotz et al. (1998), Guerinot (2000), Chiang et al. (2006), Talke et al. (2006), Yang et al. (2010), Assunção (2010)
ZIP4	Zn	Zn, Cu	<	Grotz et al. (1998), Wintz et al. (2003), Talke et al. (2006), Yang et al. (2010), Assunção (2010)
ZIP5	Zn	Zn, Mn	–	Wintz et al. (2003), Assunção (2010), Milner et al. (2013)
ZIP6	–	Mn	<	Becher et al. (2004), Milner et al. (2013)
ZIP7	–	Zn, Mn	–	Milner et al. (2013)
ZIP8	–	–	–	
ZIP9	Zn	Zn, Mn	<	Weber et al. (2004), Talke et al. (2006), Yang et al. (2010), Assunção (2010), Milner et al. (2013)
ZIP10	Fe, Zn	Zn	<	Talke et al. (2006), Assunção (2010), Milner et al. (2013)
ZIP11	Fe, Zn	Zn	–	Assunção (2010), Milner et al. (2013)
ZIP12	Fe, Zn	Zn	<	Chiang et al. (2006), Assunção (2010), Milner et al. (2013)

*Ah, A. halleri; At, A. thaliana; –, not determined.*

In *A. thaliana*, Zn toxicity causes reduced Fe uptake and shoot Fe accumulation, which indicates competition between Zn and Fe in root uptake (Fukao et al., 2011; Shanmugam et al., 2011). Excess Zn significantly reduces shoot Fe content and induces *IRT1* and *IRT2* (Fukao et al., 2011; Shanmugam et al., 2011). This response could be responsible for Zn sensitivity because IRT1 and IRT2 can also transport Zn. Zn uptake with IRT1 and IRT2 induction overloads the regular detoxification system. Interestingly, in *A. thaliana*, excess Fe alleviates Zn toxicity under excess Zn. Therefore, the competition between Zn and Fe plays an important role in tolerance to excess Zn (Fukao et al., 2011; Shanmugam et al., 2011; Pineau et al., 2012). As compared with *A. thaliana*, *A. halleri *shows altered expression of genes related to Fe homeostasis (Chiang et al., 2006; Shanmugam et al., 2011). The expression of the Fe-regulated ZIP transporters IRT1 and IRT2 is much lower in *A. halleri *than *A. thaliana *(Shanmugam et al., 2011). *A. halleri *lives in Zn-rich conditions. In *A. halleri*, high Zn concentration does not greatly affect shoot and root Fe accumulation, which could explain the reduced expression of *IRT1* and *IRT2.* Therefore, Zn uptake is mainly through Zn-regulated ZIP transporters for optimal root uptake of Zn without disturbing the expression of Fe-regulated ZIP transporters.

The high expression of ZIP transporters such as *IRT3*, *ZIP3*, *ZIP6*, *ZIP9* and *ZIP12* may also have a function in Fe availability in *A. halleri *(Chiang et al., 2006; Talke et al., 2006; Lin et al., 2009; Willems et al., 2010). The clearest example of a ZIP transporter functioning in Fe availability is *IRT3*. IRT3 can transport Zn as well as Fe (Lin et al., 2009) and is also linked to a major QTL responsible for a shoot Fe accumulation phenotype in *A. halleri* (Willems et al., 2010). In addition, by both its expression in root stele and complementing shoot Fe content in *irt1-1*, IRT3 could play a role in Fe uptake and translocation in *A. halleri* (Lin et al., 2009; Shanmugam et al., 2011). Together, the high expression of these ZIP transporters could contribute to Fe acquisition in *A. halleri *and prevent loss of control of the Fe-regulated multi-metal transporters IRT1 and IRT2 under excess Zn. The major uptake of Zn through Zn-regulated ZIP transporters in coordination with a Zn detoxification mechanism helps in Zn tolerance. Thus, the balanced control of Fe- and Zn-regulated ZIP transporters could be an adaptive mechanism in metal-rich environments.

## CONCLUSIONS AND FUTURE PERSPECTIVES

Our knowledge of metal tolerance and hyperaccumulation in plants has greatly improved in recent years with the identification of key genes and regulators involved in the metal homeostasis network. In addition to the proposed mechanisms for metal tolerance and hyperaccumulation, specificity in metal uptake could be a beneficial mechanism. Studies of the Zn hyperaccumulator *A. halleri* suggest that repression of Fe-regulated multi-metal transporters and overexpressing metal (Fe)-specific transporters may be a useful strategy for engineering plants tolerant to heavy metals. Tight control of the uptake system may also be an important strategy for tolerance of excess Zn. The function of several ZIP transporters has not been clear to date. At least, more research into the role of ZIP transporters with high expression in *A. halleri* will help in understanding their role in metal uptake and tolerance.

Apart from the transporters, several transcriptional regulators might be involved in the balanced Zn and Fe uptake in *A. halleri*, when considering the similar biological property of many ZIP transporters, but our knowledge in this area remains limited. In *A. halleri, *the major Fe deficiency regulator FIT was less regulated under Fe deficiency or Zn excess stress. This finding again suggests the occurrence of a complex process apart from what is already known in maintaining Fe homeostasis in a Zn-rich environment. In addition, the involvement of chelator complexes and their roles in facilitating the control of metal uptake and tolerance are not known. More research in these directions is needed.

## Conflict of Interest Statement

The authors declare that the research was conducted in the absence of any commercial or financial relationships that could be construed as a potential conflict of interest.
